# Biosorption of neodymium on *Chlorella vulgaris* in aqueous solution obtained from hard disk drive magnets

**DOI:** 10.1371/journal.pone.0175255

**Published:** 2017-04-07

**Authors:** Mehmet Ali Kucuker, Nils Wieczorek, Kerstin Kuchta, Nadim K. Copty

**Affiliations:** 1 Waste Resources Management, Institute of Environmental Technology and Energy Economics, Hamburg University of Technology (TUHH), Hamburg, Germany; 2 Institute of Environmental Sciences, Bogazici University, Bebek, Istanbul, Turkey; Brandeis University, UNITED STATES

## Abstract

In recent years, biosorption is being considered as an environmental friendly technology for the recovery of rare earth metals (REE). This study investigates the optimal conditions for the biosorption of neodymium (Nd) from an aqueous solution derived from hard drive disk magnets using green microalgae (*Chlorella vulgaris*). The parameters considered include solution pH, temperature and biosorbent dosage. Best-fit equilibrium as well as kinetic biosorption models were also developed. At the optimal pH of 5, the maximum experimental Nd uptakes at 21, 35 and 50°C and an initial Nd concentration of 250 mg/L were 126.13, 157.40 and 77.10 mg/g, respectively. Analysis of the optimal equilibrium sorption data showed that the data fitted well (*R*^*2*^ = 0.98) to the Langmuir isotherm model, with maximum monolayer coverage capacity (*q*_*max*_) of 188.68 mg/g, and Langmuir isotherm constant (*K*_*L*_) of 0.029 L/mg. The corresponding separation factor (*R*_*L*_) is 0.12 indicating that the equilibrium sorption was favorable. The sorption kinetics of Nd ion follows well a pseudo-second order model (R^2^>0.99), even at low initial concentrations. These results show that *Chlorella vulgaris* has greater biosorption affinity for Nd than activated carbon and other algae types such as: *A*. *Gracilis*, *Sargassum sp*. and *A*. *Densus*.

## Introduction

Neodymium (Nd) is one of the critical rare earth elements (REEs). The neodymium-iron-boron (Nd-Fe-B) alloy has been used since 1980s in the permanent magnets of numerous electronic devices [1]. The Nd-Fe-B magnets are particularly employed in hard disk drives (HDD), hybrid car engines and wind turbines [1]. Characterization of Nd–Fe–B magnets from HDD indicated that its metal composition consists of: Fe (67.4%), Nd (24.3%), Dy (1.1%), Ni (0.8), B (1.2%), Co (0.9%) and Al (0.8%).

A number of methods have been used to recover REEs from aqueous solution, most notably: chemical precipitation [2], ion exchange [3], adsorption [4–7]. In recent years, there has been growing interest in the recovery of REE through biosorption, a rapid, reversible, economical and ecofriendly technology compared to conventional methods [8]. Biosorption has emerged as a promising technology [9–15] as it encompasses the combined effects of adsorption, ion exchange and micro precipitation onto functional groups of inactive cell walls of biological origin [15–16].

This study focuses on the use of the green algae *C*. *vulgaris* as a biosorbent. Various studies have evaluated the effectiveness of *C*. *vulgaris* for the recovery of precious metals. Hosea et al. [17] and Ting et al. [18] reported that green algae *C*. *vulgaris* could be used for gold recovery from diluted solution by 90%. Immobilized *C*. *vulgaris* has also been studied as biosorbent for the recovery of precious metals Platinum (Pt) and Palladium (Pd) [19].

The purpose of this current study is to evaluate the biosorption process for the removal of Nd from a mixed leachate solution derived from permanent Nd-Fe-B magnets. We specifically examine the impact of different operational parameters on the efficiency of biosorption of Nd on *C*. *vulgaris*. An important feature of this study is that the Nd aqueous solution was derived from real Nd-Fe-B magnets. Previous studies have generally relied on synthetically prepared Nd solutions. The procedure followed for the preparation of the Nd solution is described in Section 2. Moreover, previous research has identified a number of parameters that can potentially influence the efficiency of the biosorption process: namely, pH, temperature, biosorbent dosage, initial metal concentration, time and agitation rate [20]. The effects of these parameters, especially pH and temperature, were investigated while optimizing the other parameters. Best-fit isotherm and kinetic models were used to assess the efficiency of *Chlorella vulgaris* relative to other biosorbents on the recovery of Nd in aqueous solution from permanent hard disk magnets.

## Materials and methods

### Microalgae cultivation and biosorbent preparation

The microalgae *C*. *vulgaris* used in the biosorption experiment was obtained from the Culture Collection of Algae (SAG) of the Georg-August-Universität Göttingen under the serial designation SAG 211–12 (SAG, 2013). The cultivation of *C*. *vulgaris* was performed in a pilot plant of Hanse Werk AG in Hamburg-Reitbrook (53° 28´ 12´´ N; 10° 10´ 40´´ E). The detailed information about microalgae cultivation using outdoor photobioreactors (PBRs) was given by Wieczorek et al. (2014) [21]. For the biosorption experiments, the harvested microalgae was first oven dried overnight (24 hrs) at 90°C. The particle size of the dried microalgae was determined using Retsch Sieving Systems and was found to be 250 μm. The dried biosorbent dosage used in the batch biosorption experiments ranged from 0.5 to 3 g/L.

### Aqueous solution from Nd-Fe-B harddisk magnets

The Nd-Fe-B magnet samples were crushed and subsequently milled using the Retch 300 miller. The milled magnets were then sieved through a 250 μm sieve in order to obtain particles < 250 μm. The magnet powders were subjected to a roasting process which consisted of heating up to 950°C for 7 h to get full conversion of the metal into oxides [22]. The Nd solution was prepared by dissolving a roasted sample in concentrated nitric acid. A total of 150 g of Nd-Fe-B magnets was used for the experiments.

For each 15 g (dry weight) of the roasted sample, 5 mL concentrated HNO_3_ (65%) and 10 mL deionized water were added in a flask and placed onto a heating source of 80±2°C with constant stirring for 72 hours [23]. The digestate was centrifuged for 10 min at 3000 rpm and filtered through Whatman No. 41 filter paper and then through Whatman 0.45μm pore size membrane filters. Afterwards, the aqueous phase was collected in a volumetric flask. After cooling the leachate was analyzed through inductively coupled plasma optical emission spectrometry (ICP-OES) in order to determine its metal concentrations. A pink colored solution of pH 2.4 was obtained with no iron detected. The initial Nd concentration of the leachate was used as a reference. Therefore, the leachate solution was standardized at 1000 mg/L Nd. Fe, Dy, B and Co concentrations were also analyzed in the stock and in the particular concentration solutions.

### Biosorption experiments

**Fig 1:**
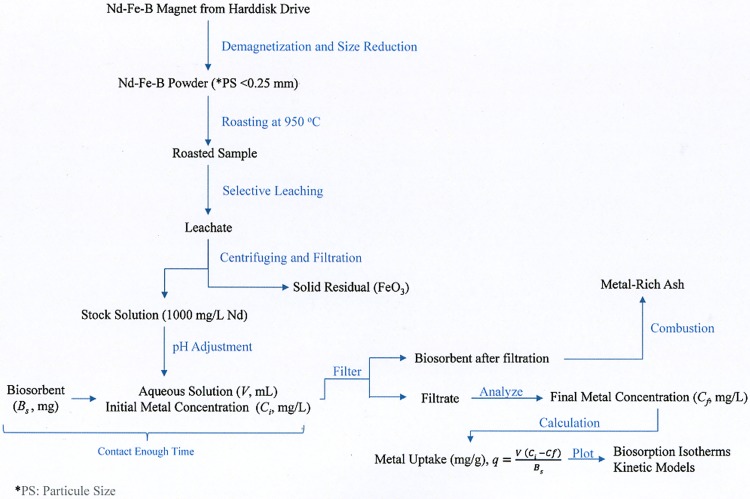
Summary of the experimental procedure.

The experiments were carried out in 500 mL Erlenmeyer flasks containing the respective amounts of sorbent dosage and stock solution. The initial Nd concentration of the aqueous solution ranging from 50 to 250 mg/L was adjusted by dilution with deionized water. The appropriate pH was regulated and kept constant within ± 0.2 deviations using 1 M HCl and 1 M NaOH solutions. Following pH adjustment, 10 mL of the sample solution was taken in order to determine the initial Nd and Fe concentrations. The same solution was also used to perform biosorption tests with 0.5–3 g/L biosorbent *Chlorella vulgaris* dosage. The dry biosorbent was contacted with 0.2 L of known concentration solutions for 90 min and the suspension was agitated on a rotary shaker at 300 rpm at three different temperatures (21±1, 35±1 and 50±1°C). In the batch biosorption experiments, the initial solution pH was varied between 3 and 5; this initial pH range was selected according to previous studies that reported that no lanthanide precipitations were experimentally found in aqueous solution [20]. Samples were taken with an automatic pipette at pre-determined time intervals (0, 3, 8, 15, 30, 45 and 90 min) to determine the residual metal ion concentrations in the solution. Each sample was first filtered by Whatman No. 41 filter paper, followed by Whatman 0.45μm pore size membrane filters in order to analyze the samples using ICP-OES. In addition, the biosorbent after biosorption process can be properly combusted to obtain metal-rich ash [24]. The diagram of the experimental study is shown in Fig 1. All experiments were performed in duplicates and the data presented are the average values of the two experiments.

A solid–liquid sorption system is usually conducted with two types of assessment: equilibrium batch sorption tests and dynamic sorption studies [25–26]. In the biosorption process, the metal ions in the solute bind onto the biosorbent until equilibrium is achieved. Experimental uptake of the each metal ion was calculated using the following equation:

$$q_{exp}=\frac{V\;\left(C_{i}-C_{f}\right)}{W\;}$$ (1)

where *q*_*exp*_ is the experimental equilibrium uptake (mg/g); *C*_*i*_ and *C*_*f*_ are the initial and equilibrium metal concentration in the solution (mg/L), respectively; *V* is the solution volume (L); and *W* is the dry weight of biosorbent (g).

### Modeling of biosorption isotherms and kinetics

Two equilibrium isotherm models were used to describe the experimental adsorption capacity of the adsorbents in batch mode, namely: the Langmuir isotherm which is based on monolayer adsorption of solute, and the Freundlich isotherm which was developed initially for heterogeneous surfaces [11, 27].

The Langmuir isotherm has been used to describe various adsorption processes including heavy metal sorption onto biosorbents [28]. The model has three main assumptions: i) adsorption is limited to monolayer coverage; ii) all surface sites are alike and can accommodate only one adsorbed atom; iii) the ability of a molecule to be adsorbed on a given site is independent of its neighboring sites occupancy [28]. Based on these assumptions and a kinetic principle, Langmuir proposed the following expression [29]:

$$q_{exp}=\;q_{max}\frac{K_{L}C_{e}}{1+K_{L}C_{e}}$$ (2)

where,

*q*_*exp*_ = experimental ion uptake coefficient (mg/g)

*q*_*max*_ = maximal ion uptake coefficient (mg/g)

*C*_*e*_ = equilibrium or final concentration in the solution (mg/L)

*K*_*L*_ = concentration constant needed to reach half of maximal binding (L/mg)

In the Langmuir model, the *q*_*max*_ is associated with the saturation of a fixed number of identical surface sites and, accordingly, the temperature should not have any impact on *q*_*max*_ [28]. The Langmuir constant (*K*_*L*_) can be used for determining the suitability of the adsorbate by using a dimensionless constant, commonly known as the separation factor (*R*_*L*_) which is defined as [30]:

$$R_{L}=\frac{1}{1+K_{L}C_{o}}$$ (3)

where *C*_*o*_ is the initial concentration. The adsorption process is considered unfavorable if *R*_*L*_ >1, linear if *R*_*L*_ = 1, favorable if 0< *R*_*L*_ <1 and irreversible if *R*_*L*_ = 0 [31].

The Freundlich isotherm is an empirical equation which is the most widely used isotherm for the explanation of adsorption equilibrium [27]. The Freundlich isotherm assumes that an infinite number of sorption sites can occur and that sorption can infinitely increase with increase in the aqueous concentration. The Freundlich isotherm can be expressed as [32]:

$$q_{exp}=K_{F}C_{e}^{1/n}$$ (4)

where *q*_*exp*_ is the ion uptake coefficient (mg/g), *K*_*F*_ is the Freundlich constant, *C*_*e*_ is the equilibrium concentration (mg/L), and *n* is the Freundlich power. In the special case when *n* = 1, the Freundlich isotherm reduces to the linear isotherm.

Kinetic models are capable of identifying the adsorption mechanism type in a test system, and its potential rate-controlling steps that include mass transport and chemical reaction processes [33]. In addition, kinetics studies are necessary to define the optimum condition for full-scale batch metal removal processes [33]. The pseudo-first- and pseudo-second-order models are most commonly used for biosorption kinetics of heavy metals and the calculation of metal uptakes [27].

The pseudo-first-order model can be written as [34]:

$$q_{t}=\;q_{exp}\left(1-e^{-k_{1}t}\right)$$ (5)

The pseudo-second-order model can be written as [35]:

$$q_{t}=\;\frac{q_{exp}^{2}k_{2}t}{1+q_{exp}k_{2}t}$$ (6)

where, *q*_*exp*_ is the amount of metal uptake at equilibrium (mg/g); *q*_*t*_ is the amount of metal uptake at time *t* (mg/g); *k*_*1*_ is the pseudo-first order rate constant (1/min); *k*_*2*_ is the pseudo-second order rate constant (g/mg-min).

The time-dependent removal percentage was calculated for different Nd concentrations and for variable sorbent dosages at different pH values. In order to gain further insight of the multicomponent biosorption uptake at different pH values, SIGMAPLOT software was used for statistical analyses and for plotting the theoretical adsorption isotherms. The Langmuir and Freundlich best-fit constants were calculated using the linearized forms of the models [36].

## Results and discussion

Different experimental conditions were assessed in order to determine the optimal conditions for biosorption of neodymium by *C*. *vulgaris* and high uptake value of Nd from harddisk magnets. The experimental results of this study are presented in this section.

### Effect of temperature, initial pH, initial Nd concentration and biosorbent dosage on Nd biosorption

**Fig 2:**
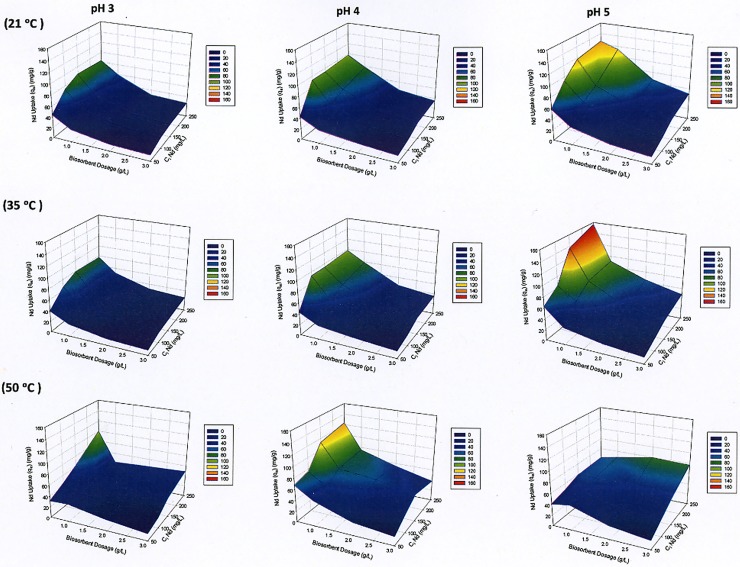
Experimental Nd uptake onto C. vulgaris at different temperature (21, 35 and 50°C) and pH (3, 4 and 5).

The biosorption experiments of Nd from hard disk magnets on *Chlorella vulgaris* were performed at three different pH values (3, 4 and 5) and three temperature (21, 35 and 50°C) for different biosorbent concentrations (0.5, 1, 2 and 3 g/L) and initial Nd concentrations (50, 100, 150 and 250 mg/L). The entire equilibrium performance of metal sorption is graphically displayed in terms of a series of 3D plots (Fig 2). The experimental neodymium uptake values onto *C*. *vulgaris* are plotted as a function of biosorbent dosage and initial metal concentrations. The grid surface which is an interpolation between the individual points [37] shows the influence of initial pH, initial Nd concentration and biosorbent dosage at the three considered temperatures: 21, 35 and 50°C.

Previous research has shown that the solution pH is an important factor controlling biosorption of REEs [11, 14, 38, 39]. The pH affects not only the biomass site dissociation, but also the solution chemistry of the metals [40–41]. Vijayaraghavan et al., [39] reported that as the H^+^ ions concentration decreases, the attraction between positively charged lanthanide ions and negatively charged binding sites of the biomass increases, resulting in an increase in removal efficiency. At low pH value there is strong competition between hydrogen ions and metal ions for active sites [38]. Romera et al. [42] conducted a series of biosorption tests using six different algae at pH values between 2 and 6 and reported that the pH affected biosorption efficiency on all six algae similarly, with higher electrical attraction to positively charged metal ions occurring at higher pH values.

Comparison of the different biosorption results indicates that the solution pH is a critical factor influencing Nd biosorption. The optimum pH value for Nd biosorption at 21 and 35°C was pH 5. At 50°C, the maximum experimental uptake (*q* = 123.82 mg/g) was obtained at pH 4, since a higher pH coupled with increase in temperature leads to Nd precipitation. Therefore, the decrease of metal ion concentration in solution resulted in low experimental Nd uptake by microalgae. On the other hand, the leachate solution derived from hard disk permanent Nd-Fe-B magnets involves mainly Nd^3+^ and Fe^3+^. Hence, strong competition occurs between Nd and Fe in order to bind to the negative functional group on the cell wall of microalgae during biosorption experiment. When the pH increased from 2 to 5, iron ions started to precipitate which leads to a decrease in the competition. However, as discussed in Section 2.2, a selective leaching procedure was applied in order to prepare an aqueous solution without iron from the Nd-Fe-B magnets. According to Takeno [43] Fe^3+^ is present at a pH between 0 and 3.8 while Fe^2+^ can be found between pH 0 and 9. The electrochemical reaction from Fe^2+^ to Fe^3+^ in aqueous solution is spontaneous but slow and difficult without an oxidant (e.g. oxygen gas) [22]. Thus, HNO_3_ was used in the solution preparation (Section 2.2) in order to react electrochemically with Fe^2+^ to produce Fe^3+^. Analysis of the stock and the particular concentration solutions indicated that Fe, B, Dy and Co concentrations were below the detection limit because of the selective leaching. Thus, these metals did not influence the biosorption tests. Moreover, most Lanthanides including Nd are in ionic form at acidic to neutral pH and Nd does not form any hydroxide precipitation at a pH lower than 7 [43].

A rising trend in Nd uptake was observed with increase in pH at 35°C. A pH 5 had the best experimental uptake (*q*_*exp*_ = 157.40 mg/g) due to less competition of H^+^ protons and a more favorable *pK*_*a*_ for the carboxylic acid groups. A pH of 4.8 had been determined as optimal for mono-metal uptake since this is the *pK*_*a*_ of the carboxylic acid functional group that is responsible for the metal immobilization in the biosorption process by 90% [39, 44, 45, 46].

Temperature plays also an important role in metal adsorption. Change in temperature affects a number of factors such as the stability of initial metal concentration in solution and the stability of the negative functional group on the biosorbent [47]. Results of the biosorption tests (Fig 2) indicated that temperature does not significantly increase biosorption in the range of 20–35°C at pH 3 and 4. At pH 5 with biosorbent dosage of 0.5 g/L at 35°C, the maximum Nd uptake value (*q*_*exp*_) is 157.40 mg/g. On the other hand at 50°C, the maximum experimental Nd uptake value is 79.51 mg/g (Fig 2). It was reported that the active binding sites in the biosorbent are affected by the high temperature, leading to lower metal uptake [48]. In addition, the equilibrium constant decreases with increasing temperature, since simple physical sorption processes are generally exothermic [49]. Similar results have been published for the biosorption of heavy metals on activated sludge [50]. The maximum adsorption capacity of sludge was found when the temperature decreased due to the fact that biosorption is an exothermic process [50]. In another study Fujiwara et al. [51] investigated the effect of temperature on adsorption of Pt, Pd and Au ions onto l-lysine at 30, 40 and 50°C and reported that the adsorption capacity decreased with increasing temperature which was also attributed to the exothermic nature of the adsorption process.

Another important operational variable influencing metal uptake is the biosorbent concentration. The maximum biosorption capacity corresponded to the lowest biosorbent dosage (0.5 g/L) at 21 and 35°C. A common trend that can be observed for all temperature and pH values is that a low biosorbent dosage combined with a high initial Nd concentration yields the highest Nd uptake. Kucuker et al. [20] reported that an increase in sorbent dosage was correlated with an increase in removal rates but also a decrease in removal efficiency for Nd from aqueous solution. An increase in biomass led to interference between the binding sites [52]. An increase in the biosorbent concentration theoretically increases the amount of solute biosorbed because of increased the number of binding sites. On the other hand, the metal uptake decreases with increasing biosorbent dosage which may be due to complex interaction of several factors [53] such as the insufficiency of the available solute to completely cover the available exchangeable sites on the biosorbent [54] and high biomass concentrations as it can exert a shell effect by protecting the active sites from being occupied by metal [42]. Therefore, a low biosorbent concentration with a higher Nd uptake can be attributed to the relatively high ionic concentration in the solution which can maximally bind to functional groups of the biosorbent [55, 42].

### Biosorption isotherms

The Langmuir and Freundlich isotherms were fitted to the Nd biosorption data. The best-fit parameters of the Langmuir and Freundlich isotherms and their correlation coefficients (*R*^*2*^) are shown in Table 1. The *R*^*2*^ values are comparatively high at 21 and 35°C compared to the data at 50°C for 90 mins biosorption test. The higher *R*^*2*^ values for the Langmuir isotherm (0.638–0.9924) compared to the *R*^*2*^ values of the Freundlich isotherms (0.547–0.877) suggest that the Langmuir model is well suited for predicting the maximum neodymium uptake value. Furthermore, this result suggests that Nd biosorption onto *C*. *vulgaris* follows the monolayer adsorption (the surface adsorption) model. At 50°C, the low *R*^*2*^ values may indicate that the removal of Nd from aqueous solution occurred not by adsorption mechanism, but through solid precipitation. The maximum monolayer coverage capacity (*q*_*max*_) of the Langmuir isotherm model at 35°C and pH 5 was determined to be 188.68 mg/g, while the Langmuir isotherm constant *K*_*L*_ was 0.029 L/mg with the separation factor, (*R*_*L*_ = 0.12), indicating that Nd biosorption on *C*. *vulgaris* is favorable.

**Table 1:** Isotherm best-fit constants and correlation factors (*R*^*2*^) for Nd biosorption.

Temperature (°C)	pH	Biosorbent Dosage (g/L)	Freundlich			Langmuir	
			*1/n*	*K*_*F*_	*R*^*2*^	*q*_*max*_ (mg/g)	*R*^*2*^
21	3	0.5	0.32	17.30	0.774	113.64	0.946
		1.0	0.46	5.77	0.957	82.64	0.998
		2.0	0.37	5.14	0.772	44.84	0.939
		3.0	0.32	5.93	0.904	31.65	0.996
	4	0.5	0.44	10.68	0.768	166.67	0.920
		1.0	0.48	6.70	0.926	107.53	0.993
		2.0	0.32	8.12	0.944	42.19	0.952
		3.0	0.31	6.99	0.869	33.67	0.872
	5	0.5	0.35	21.72	0.930	151.52	0.996
		1.0	0.55	7.63	0.980	140.85	0.987
		2.0	0.57	4.69	0.989	90.09	0.999
		3.0	0.52	5.02	0.936	60.98	0.968
35	3	0.5	0.34	13.89	0.822	105.26	0.961
		1.0	0.36	7.87	0.813	62.79	0.926
		2.0	0.43	3.44	0.870	44.25	0.957
		3.0	0.42	3.13	0.930	33.67	0.987
	4	0.5	0.36	16.41	0.632	156.25	0.837
		1.0	0.51	64.33	0.839	151.52	0.931
		2.0	0.34	8.20	0.978	44.64	0.946
		3.0	0.45	3.87	0.959	44.05	0.979
	5	0.5	0.32	25.05	0.930	188.68	0.985
		1.0	0.47	9.73	0.835	156.25	0.961
		2.0	0.53	5.46	0.985	83.33	0.999
		3.0	0.61	3.23	0.925	82.64	0.996
50	3	0.5	0.65	2.98	0.970	121.95	0.941
		1.0	0.29	11.48	0.903	55.25	0.792
		2.0	0.33	8.93	0.806	37.59	0.665
		3.0	0.55	3.64	0.818	45.05	0.809
	4	0.5	0.35	23.65	0.753	111.11	0.650
		1.0	0.12	39.34	0.848	66.67	0.694
		2.0	0.19	12.33	0.127	43.48	0.834
		3.0	0.39	7.83	0.662	57.14	0.918
	5	0.5	0.22	19.28	0.950	65.79	0.923
		1.0	0.08	41.59	0.628	81.30	0.584
		2.0	0.13	31.56	0.676	49.02	0.691
		3.0	0.18	45.86	0.533	91.74	0.405

Although the correlation coefficients of the Freundlich isotherm were lower than that of the Langmuir isotherm, examination of the best-fit data can provide some insight on the biosorption process. In particular, 1/*n*, which is an indicator of the strength of the adsorption process [56] ranged from 0.08 to 0.65. 1/*n* values between 0.1 and 1, are indicative of beneficial adsorption [56, 57]. When the values of 1/*n* are less than 1, significant adsorption occurs at low initial metal concentration; however, the increase in the amount of the initial metal concentration is less significant at higher metal concentration and vice versa [58].

Comparison of the Nd biosorption capacities observed in our experiments with the sorption capacities of other sorbents shows that the biosorption on *C*. *vulgaris* is quite favorable. The maximum Nd sorption capacities (*q*_*max*_) were reported to be 61.7, 142.76, 73.7 and 107.07 mg/g for activated carbon, *A*. *Gracilis*, *Sargassum sp*. and *A*. *Densus*., respectively [45, 46]. These values are about 30–75% of the maximum Nd sorption capacity *q*_*max*_ = 188.68 mg/g obtained in the current study.

### Biosorption kinetics and modelling

In addition to the biosorption isotherm analysis, biosorption kinetics were carried out by observing the variation of the metal uptake as function of time under different experimental conditions. The experimental kinetic data were modeled by the pseudo-first and psuedo-second order kinetics. Results of the kinetic analysis (S1 Table) indicate that the data fitted well the pseudo-second order rate equation. The pseudo-second order modeling showed that the metal biosorption is predominantly a physiochemical between the biomass and metal ions [59, 60]. In addition, the constants of the pseudo-second order model would be based on the concentrations of the ions in the solution, the pH and the temperature of the solution [35]. This finding is consistent with the equilibrium biosorption data (Section 3.1) which showed that Nd biosorption was strongly influenced by the pH and temperature. According to the literature, chemical reactions have the main role in the rate-controlling step and the best correlation coefficient values were obtained by the pseudo-second order chemical reaction kinetics [35, 60–62].

**Fig 3:**
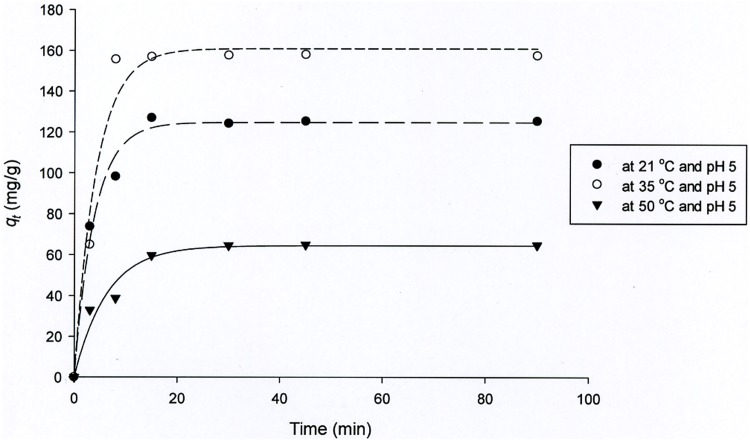
Nd biosorption kinetics for pH 5, biosorbent dosage = 0.5 g/L, initial Nd concentration = 250 mg/L and for temperature 21, 35 and 50 oC. Curves were predicted by pseudo-second order model.

Fig 3 shows Nd biosorption kinetics on *C*. *vulgaris* for the maximum uptake at 21, 35 and 50°C. The uptake generally consisted of two stages: a fast uptake rate in the first 15 min, where more than 90% of Nd biosorption occurred, followed by a slower uptake rate as equilibrium approaches. Equilibrium was reached within 30 min for all tested temperatures. The first phase is attributed to surface adsorption, mainly based on anion exchange with the participation of the carboxyl groups on the cell wall of biomass [25]. Based on the results, a pH of 5 was found as an optimum pH for biosorption of Nd, because the *pK*_*a*_ of the carboxylic acid functional group is 4.8. A number of researchers reported that the carboxylic groups play a main role for metal uptake from aqueous solution [39, 44–46].

## Conclusion

This study investigated the effect of different operation parameters, namely: the pH, temperature, biosorbent dosage and initial metal concentrations on the biosorption of Nd onto microalgae *C*. *vulgaris* biomass. The target Nd was derived from real Nd-Fe-B magnets as opposed to previous studies which have mostly used synthetically prepared Nd solutions. Results show that the optimal Nd biosorption on microalgae *C*. *vulgaris* biomass was *q*_*exp*_ = 157.40 mg/g which was observed at a temperature of 35°C, and for pH 5 and 250 mg/L Nd initial concentration for 90-min biosorption test. Analysis of the biosorption equilibrium data indicated that the data fitted well the Langmuir adsorption isotherm with the maximum sorption capacity (*q*_*max*_) calculated to be 151.51, 188.68 and 121.95 mg/g at 21, 35 and 50°C, respectively. It was also observed that the percentage removal of Nd increases with increase in biosorbent dosage and decreases with initial Nd concentration. The kinetic biosorption data indicate the Nd^3+^ biosorption is very rapid in the initial stage followed by a much slower rate until equilibrium is reached. Sorption of Nd^3+^ ion follows pseudo-second order equation with a correlation (R^2^>0.99) even at lower initial concentrations. Overall, this study demonstrates that high Nd biosorption may be achieved with *C*. *vulgaris and* provides some insight into the uptake mechanism and sorption behavior of Nd derived from Nd-Fe-B magnets on *C*. *vulgaris*.

## Supporting information

S1 Table Results of the kinetic analysis. (XLSX)
